# Emerging aspects of mobile phone use

**DOI:** 10.3134/ehtj.09.005

**Published:** 2009-06-12

**Authors:** F Samkange-Zeeb, M Blettner

**Affiliations:** Institute of Medical Biostatistics, Epidemiology and Informatics (IMBEI), Johannes Gutenberg University, Mainz, Germany

## Abstract

The mobile phone is a modern-day invention, which has managed to reach many parts of the world enabling telecommunications across areas where it was not possible before. Although these devices have proved to be life saving in certain circumstances (e.g., after accidents) and helped improve the quality of life in some sectors, concerns continue to be raised about potential adverse health impacts associated with their use. These range from cancer and cognitive deficiencies to subjective effects, such as a feeling of warmth around the ear used, headache and fatigue. We provide an overview of the concerns raised and summarise what is known about them. We conducted a literature search in Pubmed/Medline to identify published papers on health effects of mobile phones, and an intensive search on the Internet to collect data on the global use of mobile phones. In the year 2000, there were an estimated 500 million mobile phone users worldwide. Today, there are about 3.3 billion users. The use of mobile phones among young children and adolescents is also increasing. Health-risk research has mainly focused on adults and on a single outcome, brain tumours. No significant relationship has been established between mobile phone use and the incidence or growth of brain tumours. Other research indicates emerging concerns, including hearing problems and self-reported health symptoms, such as tiredness, stress, headache, anxiety, concentration difficulties and sleep disturbances, but results remain inconclusive. Currently, there is little epidemiological evidence indicating that the use of mobile phones causes adverse health effects.

## Introduction

The mobile phone is a modern-day invention, which has reached many parts of the world. Since their introduction in a few countries in the 1980s, mobile phones have gone from being expensive items that were mainly used by the business elite, to being communication tools used by the general population. The use of mobile phones is now so extensive that in some countries the number of phone subscriptions outnumbers the population.^1^ In many developing countries, mobile phone technology has made it possible for communities with scarce ‘landline’ infrastructure to participate in telecommunication.

Although mobile phone use has proved to be life saving in certain circumstances (e.g., after accidents) and has helped improve the quality of life in some sectors, concerns continue to be raised about potential adverse health impacts associated with their use. As mobile phones are generally held close to the head during use, one of the concerns widely expressed has been that the radiofrequency (RF) waves emitted during use might lead to an increase in brain cancer risk.^2^ To date, a substantial number of epidemiological studies on the possible link between mobile phone use and increased brain tumour risk have been published, but no clear positive association has been found.^3^–^18^ Some uncertainty, however, still remains, particularly regarding long-term effects of mobile phone use (≥15 years).

Other health concerns associated with mobile phones range from cognitive deficiencies to subjective effects, such as a feeling of warmth around the ear used, headache and fatigue.^19^ In some countries, it is now forbidden to use mobile phones while driving, as this has been shown to distract drivers, leading to accidents.^20^–^22^ Effects of mobile phone use in childhood have yet to be investigated adequately.

Our main aim in this paper is to give a broad outline of the health concerns that have arisen from mobile phone use, and to summarise what is known about these adverse effects. We will focus not only on effects that have been investigated extensively, such as brain cancer, but also on some outcomes that are currently discussed as being of potential concern (e.g., hearing and behavioural problems), which have not yet been studied in population-based studies.

A brief outline of the global prevalence of mobile phones will be followed by a short summary of current epidemiological findings on the potential link between mobile phone use and brain tumour risk. Some of the problems, challenges and limitations of the epidemiological studies will also be discussed. In the second part of the paper, we will look at the health issues associated with mobile phone use that might be of interest for future research, also focusing on potential adverse effects of mobile phone use in childhood.

### 

#### Some aspects of RF exposure

Mobile phones are not the only source of RF fields to which the public is exposed. Non-occupational exposure can come from radio and television transmitters, or through cordless phones. The intensity of the RF fields emitted, and not just their presence, is important in terms of potential health effects.^23^ The specific energy absorption rate (SAR) is a measure commonly used to calculate the RF energy absorbed by the body during mobile phone use. Mobile phones typically operate at frequencies of 450–900 MHz (analogue systems), 1800–1900 MHz (digital systems) and 1900– 2200 MHz (Universal Mobile Telecommunications System).^23^ The highest brain SAR values measured in a laboratory using real mobile phones were reported to be in the range of 0.9–1.76 W/kg for analogue phones, and 0.44 W/kg for digital Global System for Mobile Communications (GSM) phones—values that fall below the International Commission for Non-Ionising Radiation Protection (ICNIRP) recommendation of 2.0 W/kg.^24^

#### Literature search

We searched for all studies on mobile phone use and brain tumours in PubMed/Medline using various combinations of the terms mobile phone, epidemiology, brain tumour, case–control study, cohort study, electromagnetic waves, RF and microwave. Substituting ‘cellular phones’ for ‘mobile phones’ did not yield any new items. We then selected original publications and review articles on mobile phone use and brain tumours only. To identify studies on non-cancer effects of mobile phone use, we searched the database using various combinations of the terms mobile phones, electromagnetic fields (EMFs), RF, microwave, health effects, adverse effects, subjective symptoms, epidemiology, unspecific health complaints, hearing impairment and childhood exposure. Original reports of research surveys, experimental studies and review articles were selected. We also used the search engine ‘Google’ to look for data on the global prevalence, trends and uses of mobile phones, citing some newspaper articles and reports identified during the search. Non-English language articles with abstracts written in English were also considered.

### Prevalence of mobile phone use

Mobile phones were introduced to a few markets in the 1980s, and their use only spread in the mid-1990s. However, by early 2000, there were an estimated 92 million subscribers in the United States and 500 million worldwide.^3^, ^4^ In the 15 members of the European Union before 2004, there were an estimated 344 million subscribers (out of a population of 380 million) by September 2004.^25^ The largest growth rate of mobile phone subscribers globally is currently to be observed in Africa, where an annual increase of 58% has been reported.^26^, ^27^ In May 2008, over 3.3 billion mobile phone subscribers, more than half of the world's population, were reported worldwide.^28^ It is expected that about 85% of the world's population will have access to mobile phone technology by 2010.^29^

#### Prevalence of mobile phone ownership and use among children

In many countries, especially industrialised ones, the number of young people (6–19 years old) who report having access to or owning a mobile phone is increasing from year to year (see Table 1). More than 90% of 333 children from age 6–13 years sampled in an Australian survey reported using their parents’ mobile phones sometimes, whereas 36% of those aged 10–13 years had their own mobile phones.^30^ In a Swedish study, 57.8% of 10-year-olds and 95% of 14-year-olds reported owning a mobile phone.^31^ By 2006, ownership in the UK was estimated at 40% among children 5–10 years of age, and about 90% among those aged 11–16 years.^32^ In Japan, about a third of sixth graders and 60% of ninth graders are said to own mobile phones.^33^ There are no figures available for prevalence of mobile phone use and/or ownership among children in developing countries. As the prevalence among adults worldwide has soared in the past few years, it can be assumed that exposure to the technology, if not ownership, will also increase with time among children and adolescents.

**Table 1:** Prevalence of mobile phone use among children in different countries

Country	Sweden
Observation	In a study conducted by Soderqvist *etal.* (2007) in Sweden, 79.1% of children aged 7–14 years who responded to a questionnaire reported having mobile phone access, with 57.7% reporting owning a mobile phone. In all, 49.1% of 7-year-olds reported having access to a mobile phone, but only 7.3% in the age group reported owning one. Ownership increased with age, with 57.8% of 10-year-olds and 95% of 14-year-olds owning a mobile phone
Source	Söderqvist *et al*.^31^
Country	Hungary
Observations	Of 1301 fourth grade school children who took part in a mobile phone use survey in three Hungarian cities, 76% reported owning a mobile phone. In all, 97% of the participants were either 10 or 11 years old. A small proportion (1%) of the school children reported having received their mobile phones four years earlier, at age six or seven, and 6% had received mobile phone three years earlier
Source	Mezei *et al*.^79^
Country	Germany
Observations	Prevalence of use among 12–13-year-olds increased from 3% in 1998 to 57% in 2001, 69% in 2002 and to 85% in 2007. The prevalence of use among adolescents aged 14–15 years, 16–17 years and 18–19 years rose from 6, 6 and 16%, respectively, in 1998 to 95, 96 and 96%, respectively, in 2007. In all, 95% of girls compared with 92% of boys had mobile phones
Source	Medienpädagogischer Forschungsverbund Südwest: http://www.mpfs.de">www.mpfs.de, http://www.mpfs.de/fileadmin/JIM-pdf07/JIM-Studie2007.pdf^80^
Country	UK
Observations	By 2006, two thirds of all children aged 5–16 years owned a mobile phone. Ownership was estimated at 40% among those aged 5–10 years and at about 90% for those aged 11–16 years
Source	Childwise: http://www.childwise.uk, http://www.childwise.co.uk/trends.htm^32^
Country	Australia
Observations	According to survey data, 36% of children aged 10–13 years owned a mobile phone by April 2003. Over 90% of children aged 6–9 years had used a mobile phone, usually belonging to the parents
Source	McNair Ingenuity Research: www.mcnairingenuity.com, http://www.mcnairingenuity.com^30^

### Epidemiological studies on mobile phone use and brain tumour risk

The main concern raised about mobile phone use has been that the electromagnetic waves emitted during use might increase the risk of developing brain tumours. As of 30 June 2008, 28 epidemiological studies investigating potential brain tumour risk due to mobile phone use had been published (in English) in PubMed/Medline.

#### First-generation studies

The first epidemiological studies on mobile phone use and brain tumour risk were conducted in the USA, Sweden, Denmark and Finland. A review of those studies, published between 1996 and 2002, was published in 2002.^34^ The authors reviewed three cohort and eight case–control studies, none of which showed a significant association between digital mobile phone use and malignant brain tumours. Only one out of five studies showed a significant association between use of analogue phones and acoustic neuroma.^5^ A common limitation of these early studies is the lack of long-term mobile phone users among the participants, as the technology was still relatively new. Several studies also had small numbers of participants and limited exposure assessment that was at times based only on subscriber information.^6^, ^7^ Where only subscriber information was available for participants, patterns of use could not be determined, and exposure assessment was complicated by the fact that the subscriber was not necessarily the user.

#### Second-generation studies

In an effort to overcome some of the limitations listed above, such as small numbers of participants, ‘INTERPHONE’, an international collaborative case–control study co-ordinated by the International Agency for Research on Cancer in Lyon, was initiated in 1999. Thirteen countries took part in the study (Australia, Canada, Denmark, Finland, France, Germany, Israel, Italy, Japan, New Zealand, Norway, Sweden and the UK), which aimed to recruit >7000 people with brain tumours. To date, seven individual countries have published some of their results,^8^–(16) and two combined publications have used data from five countries (see Table 2).^17^, ^18^ Comparing mobile phone users with non-users, none of the INTERPHONE publications reported an association between mobile phone use of <10 years and increased risk of brain tumours. Increased risks for regular mobile phone use of >10 years were reported in two studies: one from Sweden and one from Germany. The Swedish paper^10^ reported increased risks for acoustic neuroma, whereas the German paper reported an increased risk for glioma.^12^ These results were not replicated in the other studies.

**Table 2:** Overview of published studies conducted within the context of the ‘INTERPHONE’ case–control collaboration study

*Study*	*Glioma*		*Meningeomae*		*Acoustic neuroma*	
			
*Category*^*a*^	*Total OR (CI)*	*≥10 years OR (CI)*	*Total OR (CI)*	*≥10 years OR (CI)*	*Total OR (CI)*	*≥10 years OR (CI)*
Denmark^8^, ^9^	0.71 (0.50–1.01)	NS^b^	0.83 (0.54–1.28)	1.02 (0.32–3.24)	0.90 (0.51–1.57)	0.22 (0.04–1.11)
Sweden^10^, ^11^	0.8 (0.6–1.0)	0.9 (0.5–1.5)	0.7 (0.5–0.9)	0.9 (0.4–1.9)	1.0 (0.6–1.5)	1.9 (0.9–4.1)
Germany^12^	0.98 (0.74–1.29)	2.20 (0.94–5.11)	0.84 (0.62–1.13)	1.09 (0.35–3.37)	—	—
England^13^	0.94 (0.78–1.13)	0.90 (0.63–1.28)	—	—	—	—
Japan^14^	—	—	—	—	0.73 (0.43–1.23)	0.79 (0.24–2.65)^c^
Scandinavia+England^17^, ^18^	0.78 (0.68–0.91)	0.95 (0.74–1.23)	—	—	0.9 (0.7–1.1)	1.0 (0.7–1.5)
Franced^15^	1.15 (0.65–2.05)	—	0.74 (0.43–1.28)	—	0.92 (0.53–1.59)	—
Norway^16^	0.6 (0.4–0.9)	0.7 (0.4–1.2)	0.8 (0.5–1.1)	1.2^e^ (0.6–2.2)	0.5 (0.2–1.0)	0.5^e^ (0.2–1.5)

Abbreviations: CI, confidence interval; OR, odds ratio.

a Estimated OR with 95% CI. ‘Total’ is for any mobile phone use (in Interphone: regular use, i.e., >1 x per week). ‘=10 years’ is for mobile phone use for 10 years or longer.

b Not stated.

c Mobile phone use =8 years included.

d Publication in French.

e Mobile phone use =6 years included.

Kan *et al*.^35^ conducted a meta-analysis to examine the effect of mobile phone use on brain tumour development risk. They investigated nine case–control studies published between 2000 and 2006, six of which were part of the ‘INTERPHONE’ study.^8^, ^10^–^13^, ^17^

After combining the findings of the nine studies, the authors did not find any increased risk of brain tumours associated with regular mobile phone use. Stratifying according to brain tumour subtype (glioma, meningioma, acoustic neuroma) among mobile phone users did not change these results. A slightly increased risk of tumour development was found when studies that involved cases with ≥10 years of mobile phone use were combined (odds ratio (OR), 1.25; 95% confidence interval (CI), 1.01–1.54). The authors performed an additional analysis in which they compared analogue with digital mobile phone use for all brain tumours and found a pooled OR of 1.22 (95% CI, 1.06– 1.41). The similarity between the two ORs (mobile phone use ≥10 years and analogue versus digital phones) led the authors to surmise that a large proportion of the association observed between mobile phone use for ≥10 years and risk of brain tumours could be explained by the confounding relationship between phone type and duration of use.

Different findings have been reported for ipsilateral use (tumour and mobile phone use on the same side of the head), with some studies reporting a significant positive association for glioma^13^, ^36^ and acoustic neuroma^11^, ^17^, ^36^ for duration of use ≥10 years.

However, patients aware of the location of their tumour may have considered that mobile phone use was a cause of its development, resulting in systematic over-reporting of phone use on the side of the head where their cancer occurred. This would also result in an underreporting of contralateral use, for which decreased risks have been reported in most of the studies.^34^, ^37^

The results of the pooled analysis of all INTERPHONE data have yet to be published. It is hoped that they will help clarify whether radiofrequencies emitted by mobile phones potentially increase the risk for brain tumours, taking the issues of ipsilateral use and duration of use into consideration.

### Changes in technology and the challenges they pose

Mobile phone technology has changed considerably since its inception, with the earlier analogue phones being replaced gradually by digital ones. The former technology operates at a higher power than the latter, emitting more electro-magnetic radiation.^23^ A considerable number of long-term mobile phone users (duration of use ≥10 years) have used both analogue and digital phones, and have thus been exposed to the power settings of both technologies. This makes the differentiation between analogue and digital phone users difficult when assessing potential health risks. As the levels of electromagnetic radiation emitted by digital phones are relatively low, it can be assumed that populations need to be followed for even longer periods of time for any changes in the risk associated with mobile phone use to be observed.

### Non-cancer EMF effects of mobile phone use

#### Subjective effects of mobile phone use

During the mid-1990s, there were many reports of head-aches, feelings of discomfort and warmth behind/around or on the ear from mobile phone users in Sweden and Norway.^38^ The majority of people with complaints had switched from an analogue Nordic Mobile Telephone (NMT) to a digital (GSM) phone, and felt the symptoms only when using digital phones. A cross-sectional epidemiological study was conducted in Sweden and Norway to test the hypothesis that digital mobile phone users experience more symptoms than analogue users.^38^ The results of the study did not confirm the hypothesis. The authors, however, did find a statistically significant association between frequency of mobile phone use and prevalence of warmth, headaches and fatigue.

Although scientific studies have not confirmed that the above symptoms are directly caused by EMFs,^39^ concern about them has not abated. Reported prevalence rates of electromagnetic hypersensitivity (EHS), which refers to the attribution of symptoms to exposure of RF EMFs, ranged from 1.5 to 10% in various population-based studies, with the majority of participants complaining of sleeping disorders and headaches.^40^–^46^

Several laboratory studies have been conducted to investigate the association between RF exposure from mobile phones and the occurrence of subjective health complaints. On the other hand, studies conducted in Saudi Arabia,^47^ Egypt,^48^ and Poland^49^ reported associations between mobile phone use and headache, fatigue, dizziness, tension, difficulties with concentration and sleep disturbances. A study conducted in the UK did not confirm these results.^50^

Röösli^19^ conducted a systematic review of studies on reports published before 2007, in which RF-EMF recognition was compared between individuals with and without EHS. After performing a meta-analysis of data from provocation studies investigating the ability to perceive low-level RF-EMF, the author concluded that the majority of EHS individuals are not able to perceive low-level RF-EMF frequency under double-blind conditions. Furthermore, no evidence was found for an association between short-term low-level exposure and the occurrence of non-specific symptoms in EHS or other individuals. A call has been made for future studies to adopt an interdisciplinary approach involving psychology, laboratory study and epidemiological disciplines together with improved personal dosimetry.^51^

#### Potential effects of mobile phone use on hearing

The global widespread use of mobile phones has also given rise to concerns over whether the EMFs emitted adversely affect hearing. So far, most studies that have addressed this potential link have been experimental. Kizilay *et al*.^52^ conducted a sham exposure-controlled experimental study with 14 adult and four newborn rats. Seven of the adults and all newborn rats were exposed to 1-h mobile phone EMF for 30 days, whereas the other seven adult rats made up the control group. Distortion product otoacoustic emissions (DPOAEs) were measured in both the exposed and control groups before and after exposure to EMF. No measurable EMF-associated changes in DPOAEs could be determined in the outer and inner ears of either adult or young rats.

Several human experimental studies have also been conducted,^53^– ^59^ in which evoked otoacoustic emissions were measured in participants with normal hearing who were exposed to mobile phone EMFs. None of the studies showed any measurable immediate adverse effects on hearing. These findings were confirmed by the European multi-centre Project ‘GUARD’, which aimed to assess potential changes in auditory function due to EMFs emitted by GSM mobile phones.^60^ The above studies, however, only looked at short-term use of mobile phones. Regarding subjective hearing problems after exposure, some studies have reported increased complaints of impaired hearing, especially among frequent users of mobile phones,^61^ whereas others did not find any association.^62^

#### Mobile phone use during pregnancy

The fact that exposure from a mobile phone kept around the hip, in a purse or in a pocket, may reach a foetus during the use of a hands-free device by a pregnant woman has been mentioned in discussions about potential adverse effects of mobile phone use. The first paper investigating this issue was published in the July 2008 issue of *Epidemiology*.^63^ Mothers of 13,159 children aged 7 years, who had been recruited to the Danish Birth Cohort early in the course of their pregnancy, completed a questionnaire regarding the current health and behavioural status of their children as well as past exposure to mobile phones. Behavioural problems were defined by the strengths and difficulties questionnaire.^64^ Increased behavioural problems were observed for children with possible prenatal exposure. The authors do call for caution in interpreting the results, as the association found may be non-causal and due to confounding.

It should also be noted that very little evidence currently exists in support of the view that EMFs from mobile phones penetrate far enough into the body to affect the foetus. SAR calculations performed to model exposure at various gestational stages were found to be below the ICNIRP reference level.^65^ Further work is needed on modelling thermal effects on the foetus as well as further investigations in other cohorts.

#### Concerns about mobile phone use by the young

It has been suggested that children are potentially more vulnerable to RF fields, as their nervous systems are still developing and their brain tissue is more conductive due to a higher water content.^66^, ^67^ However, some controversy surrounds this topic, with some governments calling for caution in mobile phone usage by the young,^68^ and others saying there is no evidence to justify restriction of usage.^69^ More so for this population than for adults, the issue of potential risks from long-term use of mobile phones remains uncertain. This point is very important for children as they are bound to have a much higher cumulative exposure to RF waves by the time they reach adulthood. They not only start using mobile phones at an earlier age compared with adults, but also tend to use them more frequently than the adults.^70^, ^71^ The possibility that exposures accrued during childhood may predispose to disease later on in life cannot be ruled out.^72^

Mobile phones are not only used for telephoning, but also for sending text messages, listening to music, playing games and watching videos, thereby exposing different body parts to electromagnetic waves. Although there is currently no evidence of significant exposures from these other forms of usage, an assessment of patterns of exposure, which takes into account the various ways in which mobile phones can be used, is needed.

#### Mobile phone use in adolescents and self-reported health symptoms

In a recent publication, Söderqvist *et al*.^73^ assessed the use of wireless telephones and self-reported health symptoms in a population-based study among Swedish adolescents aged 15–19 years. Overall, regular mobile phone users reported health complaints, such as tiredness, stress, headache, anxiety, concentration difficulties and sleep disturbances more often than less frequent users. The risk of suffering from these symptoms was higher in those reporting mobile phone use of >15 mins a day. Similar results regarding sleeping problems, stress and fatigue were found by Bader after an experimental study of 21 healthy persons aged 14–20 years with normal working/studying hours and no sleeping problems.^74^ The control group (three men and seven women) made fewer than five calls a day and/or sent five text messages a day, whereas the experimental group (three men and eight women) made >15 calls a day and/or sent 15 text messages a day. The sleep–wake profiles of both groups of participants were assessed using a 1-week actigraphy and sleep diary. After being asked about their lifestyle and sleep habits, those with excessive use of mobile phones are reported to have been more restless, to have consumed more stimulating beverages, to have had more difficulties falling asleep and to have been more susceptible to stress and fatigue compared with participants with less mobile phone use. The use of mobile phones after bedtime by school children has also been found to lead to increased levels of tiredness after 1 year.^75^ The authors of this study followed up 1656 school children with mobile phones for 1 year (average age 13.7 years in the younger group and 16.9 years in the older group). Only 38% reported that they never used their mobile phones after bedtime. Compared with those who did not use mobile phones after bedtime, the odds of being very tired were 3.9 times higher among children who used mobile phones between midnight and 3 am. The cause of tiredness is more likely to be linked to staying up late at night than to RF exposure.

#### Mobile phone use and behavioural concerns

Concerns have also been raised about non-cancer, non-EMF-related effects among children, mostly behavioural problems. However, these have not yet been explored in depth epidemiologically, and the available information is more or less anecdotal. A survey conducted in Japan compared mobile phone ownership and use between 3389 non-delinquent and 993 delinquent students who had been arrested for shoplifting, stealing motorcycles and similar unlawful activities.^76^ Fifty-seven percent of the non-delinquent students owned mobile phones, compared with 71.6% of the delinquent students. Delinquent students were found to make seven calls per day and send 42.6 mails on average, whereas non-delinquent students made 2.7 calls per day and sent 30.5 mails.

In a Finnish study, health-compromising behaviours, such as smoking, use of smokeless tobacco (snuff) and use of alcohol, were linked to increased mobile phone use, with the amount of mobile phone use increasing with increasing frequency and intensity of these behaviours.^77^ As the sale of tobacco and alcohol is legal only for people above 18 years of age, the authors surmise that mobile phones may be used to pass on information and make special arrangements about the delivery of the products or where to meet to consume them. It would be interesting to learn whether this association between mobile phone use and health-compromising behaviours can be replicated in other countries. It is also not clear whether the use of mobile phones causes health-compromising behaviour, or whether the behaviour causes (or drives) mobile phone use, or if both are related to another unmeasured variable.

#### Mobile phone base stations

Although our overview concerns primarily the health effects of mobile phone use, we mention briefly the issue of exposure from mobile phone base stations as considerable concern has and continues to be raised about them. The health concerns mentioned with respect to RF-EMF exposure from mobile phone base stations include sleeping disturbances, headaches and various non-specific health symptoms among people living in proximity to the stations.^42^–^46^

RF exposures from base stations differ from those associated with mobile phone use, mostly because they are directed at the whole body and can affect everyone. Even though exposure from base stations is more continuous by comparison, it is several orders of magnitude lower than exposure from mobile phones.^3^, ^78^

## Conclusion

Mobile phones have not only helped improve communication worldwide, especially in developing countries, they have in some instances helped save lives. For many parents, they offer an important means of keeping in touch with their children as they travel to and from school, sporting activities or meeting with friends. These and other positive aspects of mobile phones should be kept in mind when discussing the issue of adverse effects of mobile phones.

A strong link has been found between mobile phone use while driving and the occurrence of traffic accidents, resulting in some governments taking steps to ban mobile phone use when navigating traffic.

At present, there is no conclusive evidence from scientific studies and health-risk assessments to indicate that RF exposure from mobile phones and their base stations lead to adverse health consequences when exposure is below recommended reference values.^46^, ^78^ No significant relationship has been established between mobile phone use and the incidence or growth of cancer, especially brain tumours. Although there is still justification for further studies, which look at the risks of longer-term mobile phone use (≥15 years), these studies should also address a broad range of health outcomes, not only brain tumours.

One concern that merits further investigation is that of subjective symptoms. Studies have shown that such complaints are not decreasing, despite the fact that no scientific evidence has been found in support of a causal relationship between EMFs below permitted levels and non-specific health symptoms.^46^ This is a broad area of investigation that requires an interdisciplinary approach with input from psychology, laboratory studies and epidemiologic disciplines.^51^

Little is known about possible adverse effects of mobile phone use on children, especially effects that might appear later in life. Ideally, prospective cohort studies covering different age groups as well as pregnant women and capable of incorporating the rapidly changing technology and exposures should be conducted.^72^ As the use of mobile phones is now so widespread, with almost everyone in industrialised countries having access to them, further studies should focus on exposure gradients rather than exposed versus non-exposed groups.^64^ Future studies should also be planned in less industrialised countries, where hardly any investigation has been carried out to date.
